# Complete Genome Sequence of *Mycoplasma capricolum* subsp. *capripneumoniae* Strain zly1309F, Isolated from Endangered Tibetan Antelope

**DOI:** 10.1128/genomeA.00496-17

**Published:** 2017-07-20

**Authors:** Huafang Hao, Shengli Chen, Yuanguo Li, Heting Sun, Ping Zhao, Yingna Jian, Yuwei Gao, Changjiang Wu, Yongsheng Liu, Yuefeng Chu

**Affiliations:** a State Key Laboratory of Veterinary Etiological Biology, Lanzhou Veterinary Research Institute, Chinese Academy of Agricultural Sciences, Lanzhou, Gansu, People’s Republic of China; b Military Veterinary Research Institute of Academy of Military Medical Sciences, Changchun, China; c State Forestry Administration, Shenyang, China

## Abstract

*Mycoplasma capricolum* subsp. *capripneumoniae* is an important pathogen of goats that causes contagious caprine pleuropneumonia. Here, we report the complete genome sequence of *M. capricolum* subsp. *capripneumoniae* strain zly1309F, isolated from a Tibetan antelope (*Pantholops hodgsonii*) in China.

## GENOME ANNOUNCEMENT

Contagious caprine pleuropneumonia (CCPP), caused by *Mycoplasma capricolum* subsp. *capripneumoniae*, is a major infectious disease characterized by high morbidity in goats. The disease is threatening disease-free countries and has been listed by the world organization for animal health (OIE) (http://www.oie.int/fileadmin/Home/eng/Health_standards/tahm/2.07.05_CCPP.pdf). It was shown that CCPP posed a great threat to the survival of endangered Tibetan antelope (1).

*M. capricolum* subsp. *capripneumoniae* strain zly1309F was isolated from lung of a Tibetan antelope with typical CCPP symptoms, including cough, pleuropneumonia, and lung lesion, in China in 2013.

The whole genome was sequenced using the PacBio platform and resulted in a total of 657.9 Mb after filtering raw data. HGAP (2) was used for *de novo* assembly and resulted in a circular chromosome of 1,016,663 bp with a 23.67% G+C content, representing a sequencing coverage of 647×. The complete genome was analyzed by GeneMarkS (3) for gene prediction, tRNAscan-SE (4) for tRNA, rRNAmmer (5) for rRNA, and sRNA were predicted by BLAST against the Rfam database (6) and cmsearch (Version 1.1rc4). The interspersed repetitive sequences and tandem repeats were analyzed by RepeatMasker (7) and TRF (8), respectively. As a result, the genome of zly1309F contains 1,208 genes, 30 tRNAs, 6 rRNAs, and 2 short RNAs (sRNAs). Its coding density is 85.68% with an average gene length of 721 bp. There are 49 interspersed nuclear elements and 111 tandem repeats, including 1 microsatellite DNA and 100 minisatellite DNAs in the genome. The IslandPath-DIOMB (9) program was used to predict the genomics islands, PHAST (10) was for the prophage prediction, and CRISPRFinder (11) for clustered regularly interspaced short palindromic repeat (CRISPR) identification. One genomic island (653,483 to 656,868) was identified in the genome. It contains 11 genes with a total length of 3,386 bp and a 19.73% G+C content. The presence of prophage will make some bacteria obtain antibiotic resistance, enhance the environment adaptability, and improve adhesion. In this genome, 4 prophages with a total length of 166,860 bp, referring to 259 genes, were found. By using CRISPRFinder, four CRISPR units with a total length of 1,339 bp were found. The functions of the predicted protein-coding genes were annotated by performing BLAST searches based in the COG (14 December 2015), NR (17 March 2015), KEGG (2016), and Swiss-Prot (4 December 2015) databases. A total of 459 open reading frames (ORFs) could be classified into 21 functional categories in clusters of orthologous groups (COG) families. In the KEGG databases, 476 genes were annotated, of which 16 of are involved in environmental information processing, 111 are related to genetic information processing, and 153 participated in metabolism. There are 34 annotated membrane transport proteins according to BLAST searches against the Transporter Classification Database (12). The Virulence Factors of Pathogenic Bacteria database (13) was used to analyze the virulence factors. Four genes, including UTP-glucose-1-phosphate uridylyltransferase (zly1309F_GM000107), magnesium-transporting ATPase, P-type 1 (zly1309F_GM000136), ATP-dependent Clp protease ATP-binding subunit (zly1309F_GM000555), and GTP pyrophosphokinase (zly1309F_GM000711) may be associated with the virulence of this strain. This is the first complete genome of *M. capricolum* subsp. *capripneumoniae* isolated from Tibetan antelope.

### Accession number(s).

The complete genome sequence of *M. capricolum* subsp. *capripneumoniae* strain zly1309F is available in GenBank under accession number CP019061.
